# Does Work-Family Conflict Mediate the Associations of Job Characteristics With Employees’ Mental Health Among Men and Women?

**DOI:** 10.3389/fpsyg.2018.00966

**Published:** 2018-06-13

**Authors:** Vânia S. Carvalho, Maria J. Chambel, Mariana Neto, Silvia Lopes

**Affiliations:** ^1^CICPSI, Faculdade de Psicologia, Universidade de Lisboa, Alameda da Universidade, Lisbon, Portugal; ^2^Faculdade de Medicina, Universidade de Lisboa, Lisbon, Portugal

**Keywords:** work-family conflict, gender, the Job Demands-Control-Support Model, workers’ mental-health, job characteristics

## Abstract

Job characteristics are important to work-family conflict (WFC). Additionally, is well established that WFC has a negative impact on mental health. As such, this research aims to examine the role of WFC as a mechanism that explains the relationship between job characteristics (i.e., those establishing by the Job Demands-Control-Support Model) and workers’ mental health. Moreover, based on gender inequalities in work and non-work roles, this study analyzed gender as moderator of this mediation. Specifically, the relationship between job characteristics and WFC and the relationship between WFC and mental health could be stronger for women than for men. With a sample of 254 workers from a Portuguese services company, (61% males), and based on a multiple-group analysis, the results indicated that the WFC mediates the relationship between job characteristics (i.e., job demands and job control) and mental health. It was reinforced that job demands and lack of control could contribute to employees’ stress and, once individual’ energy was drained, the WFC could emerge. Ultimately, may be due to the presence of this conflict that individuals mental health’ is negatively affected. Contrary to our expectations, this relationship is not conditioned by gender (*Z*-scores were non-significant). The study results have implications for human resource management, enhancing the knowledge on the relationship between the WFC and workers’ mental health.

## Introduction

The European Mental Health Action Plan for 2013–2020 underlines that mental health is one of the top public health challenges, affecting about 25% of the population every year (World Health Organization [WHO], 2015). Mental health is considering a chronic condition resulting either from an acute, intense confrontation with a stressor, such as is the case in a post-traumatic stress disorder or from the continuing presence of a stressor which may not necessarily be intense (Houtman and Kompier, 2011). Goldberg and Hillier (1979) have aided understanding of individual mental health through somatic symptoms (e.g., headaches), anxiety/insomnia (e.g., loss of sleep), social dysfunction (e.g., social withdrawal), and depression symptoms (e.g., thoughts of the uselessness of life). One of the proposed actions that the WHO highlighted to 2013–2020 is the need to “reduce psychosocial and job-related stress, enhance stress management and introduce simple programs to promote well-being in the workplace" (World Health Organization [WHO], 2015, p. 11). Therefore, a comprehensive understanding of mental health in the workplace is sorely needed.

The Job Demands-Control and Support model (JDCS; Karasek, 1979; Karasek and Theorell, 1990) is perhaps the most extensively tested and empirically validated model which, in its simplest form, explains workers’ mental health as a function of job characteristics: job demands, job control, and job support. Empirical evidence points to high job demands negatively predicting workers’ mental health, while job autonomy and job support positively predict workers’ mental health (Luchman and González-Morales, 2013). The work-to-family conflict (WFC) defined as “a form of inter-role conflict in which the role pressures from the work domain are, to some extent, incompatible with the family domain” (Greenhaus and Beutell, 1985, p. 77), is also known for its negative impact on workers’ mental health (Frone, 2000; Michel et al., 2011; Carvalho and Chambel, 2016). Moreover, research has shown that job characteristics are relevant to the WFC emergence (Michel et al., 2011). This suggests that the WFC may have a crucial role in explaining the relationship between job characteristics and workers’ mental health.

However, studies have highlighted that men and women may also perceive how their work conflicts with their family in distinct ways (Powell and Greenhaus, 2010). In fact, the traditional “ideal worker” (Kelly et al., 2010) understands the idea of a man as the earner of the primary paycheck within the family. This idea is in line with the gender ideology, according to which, women are the primary caregivers of the family (Kelly et al., 2010). Nowadays, the increasing proportion of women in the workplace (Powell and Greenhaus, 2010) has given rise to an overload of their role, since they need to accomplish their work and family responsibilities concomitantly. In accordance with OCDE (Organisation for Economic Co-operation and Development), women in Portugal, where the present study was developed, carry out more unpaid work as compared to other European countries. Specifically, Portuguese women spend more than 5 h per day in unpaid work compared to a bit more than one and a half hours for men (European Commission, 2016). Thus, women, when compared with men, may perceive more interference of job characteristics in their family life (Grönlund, 2007; Castro, 2012; Walsh, 2013), and their mental health may be more affected by their WFC (Walsh, 2013).

By integrating the JDCS theory, we explore the effect of job characteristics on workers’ mental health by testing a moderated-mediation model, where the WFC acts as the mediator and gender as the moderator of this mediated relationship.

This is a promising study in terms of its contribution to research and practice. First, this article responds to the recommendation made by Schaufeli and Taris (2014), stating that it is important to conduct research in order to clarify the mechanisms that explain the relationship between job characteristics and mental health. By the same token, Hayes (2012) also stressed that understanding the mechanisms of the relationship between two variables is more important than establishing the relationship itself. Thus, our goal is to understand the role of the WFC in the relationship between job characteristics and workers’ mental health. To our knowledge, there are no previous studies establishing this link. A previous study by Chambel et al. (2017) examined this mediation relationship, however, it focused on a specific sample of contact-center workers, where the majority of participants were single and had no children. Thus, the authors chose to measure the work-to-life conflict instead of the WFC. In the present study, we seek to understand the work and family relationship of a sample of individuals with children and/or who are married or in a common-law relationship. Second, the results of previous studies have proven to be inconclusive as far as gender differences in the WFC (Powell and Greenhaus, 2010) are concerned. Thus, research regarding men and women and their work-family experiences are sorely needed (Grönlund, 2007). Therefore, our study may provide new insights into gender differences in the relationship between job characteristics and the WFC and between the WFC and workers’ mental health. Furthermore, it contributes to a more comprehensive line of research, focusing on mediation and moderation (Hayes, 2012). Third, this study was conducted in a Portuguese context where gender issues are particularly worrisome. In line with most European countries, 47.5 % of women in Portugal make up the labor force (PORDATA, 2017). However, the gender pay gap, i.e., the difference in average gross hourly wage between men and women across the economy, is 16.3% in Europe and 17.8% in Portugal (Eurostat, 2017). As previously mentioned, for the paid and non-paid work total, women work more than men (European Commission, 2016). Thus, it is quite clear that although women in Portugal pursue a professional career, they are still required to live up to gender expectations (Vieira et al., 2014). Furthermore, there is a lack of institutional and organizational support to balance work and family responsibilities (Matias and Fontaine, 2015). Hence, this study may provide some clarity on the gender issues and work–family relationship in Portugal, namely by offering further understanding on how to prevent the WFC and promote mental health in men and women, thus contributing to the design of more effective policies in this area. Lastly, by testing the importance of the WFC in the relationship between job characteristics and workers’ mental health, this study may extend the generalizability of the importance that both job characteristics and the WFC have on workers’ mental health. Generally, we hope to contribute to the design of interventions and policies related to workplace mental health prevention and promotion.

### Theoretical Background and Hypotheses

#### The Mediating Role of the WFC

As Luchman and González-Morales (2013) advanced, the JDCS (Karasek, 1979; Karasek and Theorell, 1990) is an influential theory for understanding how job characteristics, i.e., demands and resources (control and support) contribute to employee mental-health. Job demands (e.g., workload; time-based demands) may be understood as a perceived lack of potential loss of personal resources for dealing with job requirements (Luchman and González-Morales, 2013). Autonomy refers to the ability to make decisions about work, the ability to be creative and to use and develop new skills, or professional development (Karasek, 1979). A worker has autonomy when he/she has the opportunity to use skills and decision authority (the individual’s ability to make a decision on the work itself). Social support is understood as an interpersonal transaction that may include an emotional expression of concern, instrumental assistance, or information (Johnson and Hall, 1988). Although the JDCS model includes supervisory and co-worker support as social support since they represent workers’ social capital at work (Nahapiet and Ghoshal, 1998) the present study has only taken supervisory support into consideration, as the organization involved in this study was characterized by a well-defined hierarchy without teamwork.

The JDCS stated that jobs with low demands, high autonomy, and high support predict workers’ well-being. For example, an employee that have heavy workload but enjoy take his/her own decision about how work should be accomplished and perceived that if he/she needs his/her supervisor listen to his/her doubts and give suggestions when needed, more easily fell encouraged to face the work challenges and, consequently, the mental health is positively affected. On the contrary, jobs with high demands, low autonomy, and low support are fatal to employees’ mental health (Luchman and González-Morales, 2013).

The WFC is grounded on theories of role stress and inter-role conflict (Kahn et al., 1964) which argue that each individual has limited resources (e.g., time, energy, and attention) to spend on life roles (Goode, 1960). Conflicts occur when individuals’ work participation conflicts with family participation (Greenhaus and Beutell, 1985). Lapierre and Allen (2006) stated that WFC is evoked by the demands of the work role that, in turn, deplete individual’ resources (e.g., time, energy, emotions) required to participate in the family role. Therefore, the presence of job characteristics that exhaust workers is fatal for the WFC emergence, whereas the presence of job characteristics that help workers to face work demands are important to prevent the WFC. As such, evidence has shown that job demands are a threat to the work-family balance by promoting WFC (Michel et al., 2011). On the contrary, autonomy enables a worker to deal with the demands which, in turn, may prevent the WFC through the management of time and space devoted to work or family roles (Grzywacz and Marks, 2000). Akin to autonomy, supervisory support is related to lower levels of workers’ WFC (e.g., Kossek et al., 2011). According to these results, job characteristics may be seen as important antecedents to a myriad of WFC.

ten Brummelhuis and Bakker (2012) also acknowledge that the WFC depletes personal resources and restrains accomplishment in the family domain. This framework, in line with the conservation of resources theory (Hobfoll, 2002), defends that as a result of a loss of resources, mental-health is threatened. It is, therefore, not surprising that a number of prior empirical studies have found that the WFC is negatively related to workers’ mental health. For example, high levels of the WFC have been found to be associated with increased levels of metabolic syndrome in younger workers, in addition to shortened hours of sleep (Berkman et al., 2015). Other stress-related outcomes that have been observed to be WFC outcomes are anxiety (Frone, 2000), substance abuse (Frone, 2000), psychological ill-being (Neto et al., 2016), and poor perceived health (Carvalho and Chambel, 2016).

Taken together, in the presence of high demands, low autonomy and low support the individual’s stress arises which affects the performance of the individual in the family role. For example, the individual arrives at home strained and do not contribute to the trivial housework and, as a consequence, a marital discussion emerged. Ultimately, the individual feels that cannot afford the demands of the work and family roles which evoke thoughts of the uselessness of life or loss of sleep. Indeed, considering the effects of job characteristics on WFC and the effects of WFC on mental-health, we argue that WFC could explain the relationship between job characteristics and mental health.

Therefore, we predict that:

**Hypothesis 1:** The WFC mediates the relationship between job characteristics (i.e., job demands, job control, and supervisory social support) and workers’ mental health.

#### The Moderating Role of Gender

The moderating role of gender is of particular interest in our model in both the relationship between job characteristics and the WFC and in the relationship between the WFC and workers’ mental health. According to Powell and Greenhaus (2010), role identities are socially defined and individuals acquire beliefs about social roles expected for women and for men. Indeed, women are viewed as more family-centered and men are viewed as more work-centered (Kelly et al., 2010). Consequently, men are usually less involved in family activities (i.e., parenting; domestic tasks) (Powell and Greenhaus, 2010) whereas women accumulate more professional work and family responsibilities (Greenhaus and Powell, 2006). Furthermore, according to boundary theories, individuals tend to either segment their work and family in separate spheres or to integrate work and family domains in permeable boundaries (Powell and Greenhaus, 2010). Considering the aforementioned, women, compared to men, have to make extra efforts to adapt their daily agenda in order to keep the boundaries more permeable, which can predict high levels of the WFC (Powell and Greenhaus, 2010). Thus, we are of the opinion that the perception of job characteristics, such as autonomy and supervisory support, which allow individuals to balance their work and family spheres, will be more valuable for women than for men. On the contrary, as women tend to make more efforts to integrate work and family, job demands may be more easily depleting and give rise to a conflict in these domains. In line with the argument of Greenhaus and Beutell (1985), “segmenters” are less likely to allow job demands to interrupt the time devoted to family. In other words, the relationship between job characteristics and the WFC may be stronger for women in comparison to men. Our argument is in keeping with previous findings. For instance, the qualitative study of Castro (2012), conducted in a company in Mexico, concluded that accomplishing work time demands is viewed as a sign of masculinity, thus maintaining women connected to traditional family roles. This means that it is more acceptable for men to deal with these time demands than for women. Hence, this particular job demand more easily creates a WFC for women than for men. Using the Job-Demand Control model (Karasek and Theorell, 1990), Grönlund (2007) analyzed the differences between men and women and observed that the WFC in women is lower when job autonomy is high. In the same vein, Walsh (2013), conducting research in a hospital, concluded that the WFC affects both men and women, but women tend to depend on social support to reduce burnout and decrease turnover when compared with men. Thus, autonomy and supervisory support may be seen as crucial characteristics that enable women to balance their work and family demands.

Moreover, when the WFC occurs, it may have stronger implications for the mental health of women than of men. As mentioned above, women are more involved in family activities while also accumulating professional work (Powell and Greenhaus, 2010). Thus, the increased likelihood of role conflict and the lack of personal resources to deal with conflict (ten Brummelhuis and Bakker, 2012) may have stronger negative consequences for women than for men. This argument is also in line with previous research. For instance, the study of Walsh (2013), which explored how factors relating to the work–life interface affected the well-being of a sample of hospital doctors, concluded that female doctors were more likely to experience job burnout than men. The longitudinal study of Kinnunen et al. (2007) also suggested that there might be gender differences in the WFC and well-being across time. More specifically, their findings revealed that unlike men, the WFC perceived at time 1 by women significantly predicted job dissatisfaction, parental distress, and psychological symptoms at Time 2, 1 year later. Taking this together, we established the following hypothesis:

**Hypothesis 2:** The mediating role of the WFC is moderated by gender. This variable interacts: first, with job characteristics, so the relationship between job characteristics and the WFC is significantly stronger for women than for men; and second, with the WFC, so the relationship between the WFC and mental health is significantly stronger for women than for men.

## Materials and Methods

### Procedure and Participants

The Ethics Committee of the Lisbon Academic Medical Center approved the study and data collection protocol. This study is an observational study which was carried out before 2013 (last update of the Declaration of Helsinki). No medical experiments were conducted and no sensitive data was collected, therefore the Ethics Committee did not require a formal consent return. However, a statement of study objectives and purposes and an anonymity and confidentiality warranty were set out at the beginning of the questionnaire, along with the professional identification and contact of the main researcher. Respondents were asked to read these statements before proceeding to answer the questionnaire. They were also informed that their participation was voluntary and they could also freely participate in or give up the study anonymously, at any moment, without any further consequences.

The questionnaire link was forwarded by the researcher team to the company contact point, who sent it to respondents via e-mail, in which they were also informed of the respective board of director’s authorization to conduct the study, after a presentation of the research protocol. There was no incentive (cash or otherwise) for participating in this study. The respondents were employed at a nationwide technological and services company with a population of 631 workers. Of all the workers invited to answer the questionnaire, 312 (49.44%) responded.

Since the goal of this study was to gain an in-depth understanding of the work and family relationship of workers, the questionnaires of workers who reported having at least one child and/or who were married or in a common-law relationship and obtained 254 answers were selected: 100 (39.4%) women and 154 (60.6%) men. The mean age of the respondents was 52.83 years (*SD* = 6.29). A large percentage of the participants (44.1%) had completed secondary schooling and 29.1% of the participants had graduated from university. All respondents worked full-time. The majority of the respondents were married or cohabiting (87.0%) and had children (77.2%). As may be observed in **Table 1**, the demographic characteristics of women and men were similar. According to the Head of Human Resources, the sample characteristics are in line with the demographic characteristics of the company workers.

**Table 1:** Demographics of the sample.

Sub-sample	Women (*n* = 100)	Men (*n* = 154)
Age (mean)	*M* = 51.59	*M* = 53.63
	(*SD* = 7.61)	(*SD* = 6.24)
	min = 27; max = 62	min = 28; max = 65
**Education (%)**		
Secondary	64%	61.7%
Graduation or higher	36%	38.3%
Had children (% Yes)	74.00%	79.20%
Married or cohabiting (% Yes)	77.00%	93.50%

### Measures

#### Job Characteristics

Job characteristics were measured using the Job Content Questionnaire (Karasek et al., 1998), which was used in a previous Portuguese study (Carvalho and Chambel, 2016). Items for job demands were: workload and time pressure (7 items) – “*I have too much to do*” (women α = 0.85 and men α = 0.89); job control (4 items) – “*I have the opportunity to decide how to organize my work*” (women α = 0.85 and men α = 0.83); supervisory social support (4 items) – “*My supervisor is concerned about the welfare of those under him*” (women α = 0.91 and men α = 0.89). Items were scored on a four-point rating scale from (1) “*totally disagree*” to (4) “*totally agree.*” In order to examine the psychometric properties of the measure, we performed a CFA. First, we tested a three latent factor model (i.e., job demands, job control, and supervisory social support) through CFA and then we compared this structure with an alternative model, where all the items loaded onto a single latent factor. The three latent factor model showed an acceptable fit to the data [*women’s sample*: χ^2^(85) = 141.86, *p* < 0.001; SRMR = 0.07; IFI = 0.93; CFI = 0.93; RMSEA = 0.07; *men’s sample*: χ^2^(85) = 204.96, *p* < 0.001; SRMR = 0.07; IFI = 0.91; CFI = 0.91; RMSEA = 0.07]. The alternative tested model did not show an acceptable fit to the data, [*women’s sample*: χ^2^(87) = 523.36, *p* < 0.001; SRMR = 0.12; IFI = 0.65; CFI = 0.65; RMSEA = 0.13; *men’s sample*: χ^2^(87) = 484.07, *p* < 0.001; SRMR = 0.13; IFI = 0.58; CFI = 0.57; RMSEA = 0.22], and differed significantly from the three factor structure model [*women’s sample*: Δχ^2^ (2) = 381.05, *p* < 0.01; *men’s sample*: Δχ^2^ (2) = 279.11, *p* < 0.01], which appears to suggest that job demands, job control, and supervisory social support consist of three different and broader types of job characteristics.

#### Work-Family Conflict

We measured the WFC using 14 items from the extended scale of Carlson et al. (2000). Example items included “*After work, I am too tired when I come home to do some of the things I’d like to do*” and “*My job takes time from me that I would like to spend with my family/friends.*” The items were answered on a five-point rating scale that ranged from “*hardly ever*” (1) to “*almost always*” (5), (women and men α = 0.92). This scale has previously been validated for the Portuguese population (Vieira et al., 2014). In order to examine the psychometric properties of the measure, we performed a CFA. We tested a one-factor model, where all the items loaded onto a single latent factor. This single latent factor model showed an acceptable fit to the data [*women’s sample*: χ^2^(73) = 143.14, *p* < 0.001; SRMR = 0.06; IFI = 0.94; CFI = 0.94; RMSEA = 0.06; *men’s sample*: χ^2^(73) = 168.70, *p* < 0.001; SRMR = 0.06; IFI = 0.93; CFI = 0.93; RMSEA = 0.07), which appears to suggest that the work-family conflict (WFC) consists of one broader variable.

#### Workers’ Mental Health

This variable was measured with a Portuguese version of the General Health Questionnaire (GHQ: Goldberg and Hillier, 1979), which includes four dimensions: somatic complaints (7 items, “*Have you recently been feeling perfectly well and in good health?*,” women α = 0.74, men α = 0.82); anxiety (7 items, “*Have you recently lost sleep over worry?,”* women α = 0.90, men α = 0.91); social dysfunction (7 items, “*Have you recently been taking longer over the things you do?*,” women α = 0.84, men α = 0.82); and depression (7 items, “*Have you recently felt that life is entirely hopeless?*,” women α = 0.87, men α = 0.81). In the GHQ-28, the respondent is asked to compare his/her recent psychological state with his/her usual state. The response scale ranged from 1 (not at all) to 4 (much more than usual). This scale had previously been validated for the Portuguese population (Monteiro, 2011). The GHQ-28 is designed for the detection of non-specific psychiatric disorders among individuals in community settings. It focuses on break in normal functioning and not on permanent traits, but also considers the appearance of new and distressing experiences (Goldberg and Hillier, 1979).

In order to examine the psychometric properties of the measure, we performed a CFA. First, we tested a four latent factor model (i.e., somatic complains, anxiety, social dysfunction, and depression) through CFA and then we compared this structure with an alternative model, where mental health was considered an overarching second-order variable and somatic complaints, anxiety, social dysfunction and depression dimensions first-order latent variables (Goldberg and Hillier, 1979). The four latent factor model showed an acceptable fit to the data [*women’s sample*: χ^2^(334) = 469.76, *p* < 0.001; SRMR = 0.06; IFI = 0.92; CFI = 0.92; RMSEA = 0.06; *men’s sample*: χ^2^(334) = 643.51, *p* < 0.001; SRMR = 0.08; IFI = 0.90; CFI = 0.90; RMSEA = 0.07]. The alternative tested model also showed an acceptable fit to the data [*women’s sample*: χ^2^(346) = 468.14, *p* < 0.001; SRMR = 0.06; IFI = 0.92; CFI = 0.92; RMSEA = 0.06; *men’s sample*: χ^2^(346) = 621.57, *p* < 0.001; SRMR = 0.08; IFI = 0.90; CFI = 0.90; RMSEA = 0.07]. In the women’s sample, these two models did not differ significantly [Δχ^2^ (2) = 1.62, *p* = 0.45], however, in the men’s sample, these models differed significantly [Δχ^2^ (2) = 21.94, *p* < 0.01] and the data was observed to fit better with the model that considered a second-order variable including the four dimensions as first-order latent variables. Thus, we considered somatic complaints, anxiety, social dysfunction, and depression to consist of four different dimensions in the same broader construct of poor mental health (women α = 0.93 and men α = 0.92).

#### Control Variables

Age may be related to the WFC (Michel et al., 2011). Thus, we controlled for age (in years). It would be also important to include tenure as a control variable. However, due to confidentiality reasons, the human resources department did not allow us to collect this demographic variable. Though, we collect age. We also recognize that despite tenure and ages were time-related variables, they are distinct. However, in a typical traditional organizational where this study was conducted the workers come in early age to the company and remain a great amount of time working in the same place. This fact helps us to think that age is proportional to tenure. Thus, we control age and we think that in this vein we are controlling the work experience too – i.e., tenure.

### Statistical Analyses

As proposed by Anderson and Gerbing (1988), we followed a two-step approach to analyze our results. Structural equation modeling (SEM) and, in particular, multiple group analysis with the AMOS software package (Arbuckle, 2003) was used, first to test several measurement models through confirmatory factor analysis (CFA) and then to compare various competing structural models. In all analyses, the maximum likelihood estimation method and the covariance matrix were used.

## Results

### Measurement Models and Descriptive Analysis

Following established recommendations (Hu and Bentler, 1999), evaluation of the overall goodness of fit of the models was based on the combination of several fit indices. Models were compared based on chi-square difference tests, and on other fit indices: the standardized root mean square residual (SRMR), the incremental fit index (IFI), the Bentler comparative fit index (CFI), and the root mean square error of approximation (RMSEA). For IFI and CFI, values above 0.90 represent a good model fit, and for SRMR and RMSEA, values equal to or below 0.07 indicate a good model fit.

For each sample, we initially performed a CFA on the full measurement model (Anderson and Gerbing, 1988). This model (five-factor model) included all observed items loading on their respective latent variables (job demands, job control, supervisory social support, WFC, and workers’ mental-health). Workers’ mental-health had four indicators (observed variables resulting from the sum of the GHQ-28 subscales items): somatic symptoms, anxiety/insomnia, social dysfunction, and depression symptoms. The latent variables were allowed to correlate with each other. We then conducted Harman’s single-factor test (Podsakoff et al., 2003), which involves a CFA in which all variables are allowed to load onto one general factor (one-factor model). In addition, as we were using more than one sample, we tested whether or not components of the measurement model were invariant (i.e., equivalent) across samples of women and men. Therefore, we ran multiple group CFA on the single scales. Results indicated that, in general, items were equivalent across samples.

The one-factor model showed a poor fit to the data across samples [*women’s sample*: χ^2^(490) = 1314.89, *p* < 0.001; SRMR = 0.12; IFI = 0.65; CFI = 0.65; RMSEA = 0.13; *men’s sample*: χ^2^(490) = 1733.19, *p* < 0.001; SRMR = 0.13; IFI = 0.62; CFI = 0.62; RMSEA = 0.13]. The five-factor model obtained a good fit across samples [*women’s sample*: χ^2^(480) = 669.27, *p* < 0.001; SRMR = 0.07; IFI = 0.92; CFI = 0.92; RMSEA = 0.06; *men’s sample*: χ^2^(480) = 738.90, *p* < 0.001; SRMR = 0.07; IFI = 0.92; CFI = 0.92; RMSEA = 0.06], and all standardized regression coefficients were significant at the 0.001 level. Furthermore, in both samples, the five-factor model fit the data significantly better than the one-factor model [*women’s sample*: Δχ^2^(10) = 645.62, *p* < 0.001; *men’s sample*: Δχ^2^(10) = 994.29, *p* < 0.001]. These analyses showed that, across samples, the factor structures of the research variables were consistent with the conceptual model and also that the manifest variables loaded on the latent variables as intended.

**Table 2** shows the means, standard deviations and correlation matrix obtained separately for women’s and men’s samples. Analyzing the correlations among the studied variables we found a significant negative relationship between supervisor support and WFC for both women (*r* = -0.33, *p* < 0.01) and men (*r* = -0.24, *p* < 0.01), a significant positive relationship between job demands and WFC for women (*r* = 0.55, *p* < 0.01) and for men (*r* = 0.49, *p* < 0.01), and a significant negative relationship between job control and WFC for women (*r* = -0.35, *p* < 0.01) and for men (*r* = 0.27, *p* < 0.01). Regarding the relationship between WFC and mental health we found a significant positive for both women and men (*r* = 0.63, *p* < 0.01; *r* = 0.69, *p* < 0.01, respectively). Thus, the correlations are in the expected direction of our hypothesis.

**Table 2:** Means, standard deviations, and correlation matrix obtained separately for women (*n* = 100) and men (*n* = 154) samples.

Variables	Women		Men		1	2	3	4	5	6	7	8	9	10
			
	*M*	*SD*	*M*	*SD*	*r* for women (below the diagonal) and for men (above the diagonal)									
(1) Age	51.59	6.20	53.63	6.24	–	-0.07	-0.03	-0.05	-0.05	0.09	0.03	0.05	0.03	0.06
(2) Job demands	2.88	0.67	2.69	0.67	-0.04	–	-0.10	-0.00	0.49**	0.32**	0.39**	0.20*	0.21*	0.39**
(3) Sup. support	3.17	0.59	2.95	0.69	0.00	-0.20*	–	0.38**	-0.24**	-0.25**	-0.21**	-0.30**	-0.15	-0.30**
(4) Job control	2.85	0.65	2.77	0.64	-0.00	-0.03	0.33**	–	-0.27**	-0.15	-0.23**	-0.28**	-0.13	-0.26**
(5) WFC	2.72	0.74	2.55	0.70	0.07	0.55**	-0.33**	-0.35**	–	0.56**	0.73**	0.27**	0.42**	0.68**
(6) Somatic c.	1.07	0.50	0.72	0.47	-0.03	0.23*	-0.19	-0.21*	0.49**	–	0.72**	0.40**	0.45**	0.89**
(7) Anxiety/ins.	1.13	0.66	0.91	0.61	0.05	0.36*	-0.24*	-0.36**	0.68**	0.74**	–	0.34**	0.56**	0.91**
(8) Social dys.	0.99	0.36	0.96	0.32	-0.03	0.21*	-0.00	-0.28**	0.39**	0.46**	0.55**	–	0.29**	0.61**
(9) Depression	0.39	0.51	0.25	0.34	0.02	0.13	-0.15	-0.32**	0.48**	0.48**	0.58**	0.48**	-0	0.55**
(10) GHQ total	1.06	0.44	0.86	0.39	0.00	0.33**	-0.20	-0.34*	0.63**	0.88**	0.93**	0.73**	0.60**	–

*^∗∗^*p* < 0.01; ^∗^*p* < 0.05.*

As expected, the job characteristics in both samples were significantly related to the WFC. As predicted, the WFC was significantly related to workers’ mental health across samples.

### Structural Models

In order to test our hypotheses, we performed a multiple group analysis. This analysis was performed according to the instructions of Byrne (2010), and by using the critical ratio for the difference between parameters method. As recommended, we first tested the structural models separately for the two sub-samples (i.e., women and men), to determine whether it was a full or partial mediation test. We confronted two structural models for both samples: a full-mediation model, which included direct structural paths from job characteristics to the WFC and from the WFC to workers’ mental-health, and a partial-mediation model in which we added direct structural paths from job characteristics to workers’ mental health. The model that best fit the data for both sub-samples of workers (women and men) was then tested in a multiple group analysis, including both sub-samples analyzed at the same time, with a view to inspecting invariance across the sub-samples (baseline model). The fit of this model was then compared to an alternative model in which we constrained all coefficient paths to be equal in the women and men sub-samples (fully constrained model: fixed measurement weights and fixed structural weights). Finally, by using the AMOS 22.0 program, *Z*-scores (i.e., the critical ratios for differences between two parameters) were also calculated for the baseline model in order to ascertain whether gender differences were statistically significant for the different relations among the studied variables. A *Z*-score above +1.645 or below -1.645 would indicate that observed differences were significant at α = 0.10. A *Z*-score above +1.96 or below -1.96 would indicate that observed differences were significant at α = 0.05. A *Z*-score above + 2.575 or below -2.575 would indicate that observed differences were significant at α = 0.01.

The full-mediation model fit the data well across samples [*women’s sample*: χ^2^(511) = 710.35, *p* < 0.001; SRMR = 0.07; IFI = 0.92; CFI = 0.92; RMSEA = 0.06; *men’s sample*: χ^2^(511) = 755.58, *p* < 0.001; SRMR = 0.07; IFI = 0.93; CFI = 0.93; RMSEA = 0.06]. We then tested an alternative partial-mediation model with paths from job characteristics to workers’ mental-health. These models also provided an acceptable fit in both the women’s and men’s samples [*women’s sample*: χ^2^(508) = 707.14, *p* < 0.001; SRMR = 0.07; IFI = 0.92; CFI = 0.92; RMSEA = 0.06; *men’s sample*: χ^2^(508) = 774.41, *p* < 0.001; SRMR = 0.07; IFI = 0.92; CFI = 0.92; RMSEA = 0.06]. However, the partial-mediation model did not fit the data significantly better [*women’s sample*: Δχ^2^(3) = 3.21, *p* = 0.36] or worse than the full-mediation model [*men’s sample*: Δχ^2^(3) = -18.83, *p* < 0.001].

**FIGURE 1:**
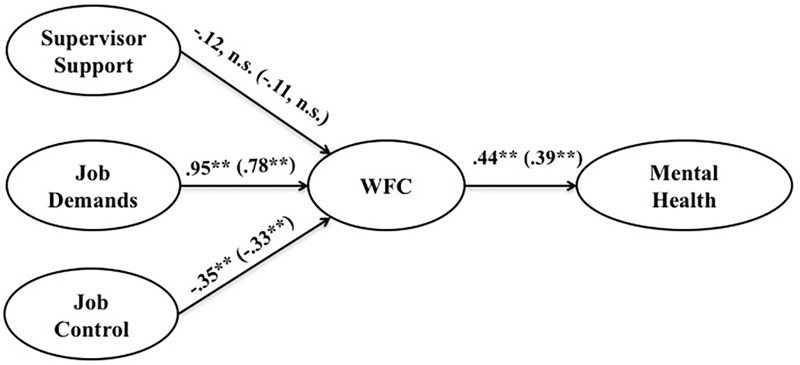
Baseline unconstrained model (*N* = 254). ^∗∗^*p* < 0.01; ^∗^*p* < 0.05; the values presented on the left side of the brackets refers to the females; the values presented within brackets refers to the males.

The full-mediation model was then tested in a multiple group analysis. We started by estimating a baseline model that included the two sub-samples at the same time, and where the structural paths were freely estimated for each group (i.e., the structural paths were unconstrained). The baseline model showed a good fit [χ^2^(1022) = 1472.88, *p* < 0.001; SRMR = 0.07; IFI = 0.92; CFI = 0.92; RMSEA = 0.04]. We then went on to constrain all the coefficient paths to analyze invariance across the groups (fully-constrained model). The fully-constrained model showed an acceptable fit to the data [χ^2^(1106) = 1612.61, *p* < 0.001; SRMR = 0.07; IFI = 0.91; CFI = 0.91; RMSEA = 0.04]. However, by comparing the fully-constrained model with the baseline model, we observed a significant drop in the fit [Δχ^2^(84) = 139.73, *p* < 0.001], which means that some coefficients were different across the two groups under study. Finally, in order to understand whether the significant differences suggested by the comparison between the fully-constrained model and the baseline model were in the relationships among the studied variables, we observed the critical ratios for differences between two parameters. The unstandardized coefficients obtained for the baseline model are presented in **Figure 1**, and in **Table 3** the *Z*-scores for the differences between the two sub-samples are presented.

**Table 3:** Unstandardized coefficients for the baseline model (*n* = 254) and *Z*-scores for differences for gender.

	Dependent variables			
	WFC		Mental health	
Gender	Females *B*	Males *B*	Females *B*	Males *B*
Supervisor support (as independent variable)	-0.12, n.s.	-0.11, n.s.	n.a.	n.a.
Job demands (as independent variable)	0.95^∗∗∗^	0.78^∗∗∗^	n.a.	n.a.
Job control (as independent variable)	-0.35^∗∗∗^	-0.33^∗∗∗^	n.a.	n.a.
WFC (as independent variable)	n.a.	n.a.	0.44^∗∗∗^	0.39^∗∗∗^

***Z*-score for differences**	**Females vs. Males**		**Females vs. Males**	

Supervisor support (as independent variable)	-0.09, n.s.		n.a.	
Job demands (as independent variable)	0.54, n.s.		n.a.	
Job control (as independent variable)	-0.12, n.s.		n.a.	
WFC (as independent variable)	n.a.		0.50, n.s.	

*WFC, work-to-family conflict; ^∗∗^*p* < 0.05; ^∗∗∗^*p* < 0.01; n.a., non-applied; n.s., non-significant.*

Hypothesis 1 predicted that the WFC would mediate the relationship between job characteristics (i.e., job demands, job control, and supervisory social support) and workers’ mental health. Regarding **Figure 1** and **Table 3**, the results obtained indicated that for both sub-samples, the relationship between supervisory social support and the WFC was not significant (for women: *B* = -0.12, n.s.; for men: *B* = -0.11, n.s.; *Z*_women-men_ = -0.09, n.s.), however, both job demands and job control were significantly related to WFC levels, namely: whereas job demands presented a positive relationship (for women: *B* = 0.95, *p* < 0.001; for men: *B* = 0.78, *p* < 0.001; *Z*_women-men_ = 0.54, n.s.), job control appeared to contribute negatively to the WFC (for women: *B* = -0.35, *p* < 0.001; for men: *B* = -0.33, *p* < 0.001; *Z*_women-men_ = -0.12, n.s.). In addition, we found that for both sub-samples, the WFC was significantly related to workers’ mental-health (for women: *B* = 0.44, *p* < 0.001; for men: *B* = 0.39, *p* < 0.001; *Z*_women-men_ = 0.50, n.s.). Thus, our hypothesis 1 was partially supported regarding the role of the WFC in explaining the relationship between job demands and job control with workers’ mental health. However, as regards the relationship between supervisory social support and workers’ mental health, we found no support for the mediating role of the WFC. As for the proposed moderating effect of gender (hypothesis 2), given that the critical ratios for the difference between parameters method (i.e., *Z*-scores) revealed non-significant group differences between women and men in the studied relationships, our hypothesis 2 was refuted by the data. Furthermore, we observed that age did not contribute to explaining variance, namely: for all the studied variables, we found non-significant relationships with age.

### Additional Analysis

Previous research (e.g., Karasek, 1998) acknowledges the possibility of the relationship between demands and outcomes (e.g., mental health) present a curvilinear relationship (i.e., an inverted U-shaped relationship). Accordingly, at a certain level demands can contribute positively to the workers’ outcomes by leading to individuals’ state of activation and energizing (Sawang, 2012; Hofmans et al., 2015). However, when demands are too high or excessive it could lead to a negative impact on workers’ outcomes (e.g., employees can feel fatigued and exhausted) (Sawang, 2012; Hofmans et al., 2015). To explore this possibility, we perform additional analyses using SPSS 25.0 version to both samples. More precisely, we used the curve estimation procedure which allows screening the data graphically to determine how the independent variable and the dependent variable are related (i.e., linearly or curvilinear). To strength the results observed by performing the curve estimation procedure, we also performed hierarchical multiple regression analysis. First, to test the main effect of demands on mental health, demands were entered at step one. Second, to determine the non-linear relationship between demands and mental health, the quadratic term (i.e., demands squared) was entered at step two (the quadratic term was calculated by squaring the appropriate continuous variable, as suggested by Aiken and West, 1991).

**FIGURE 2:**
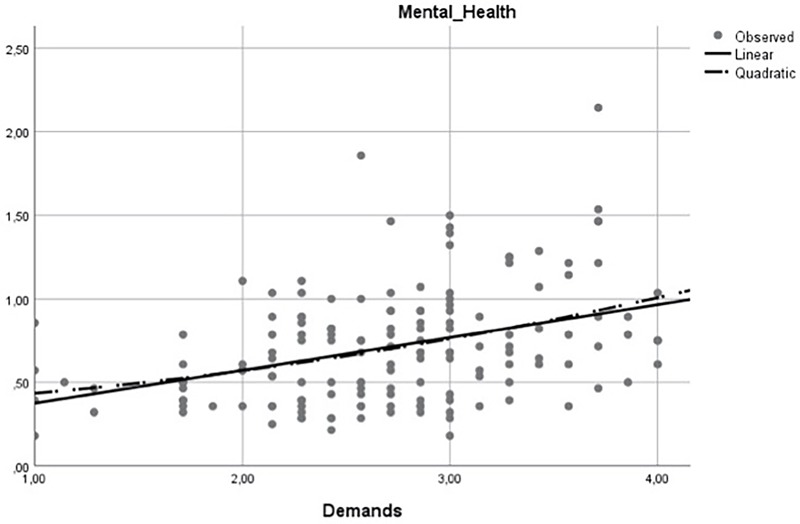
Linear and Curvilinear estimates for women.

**FIGURE 3:**
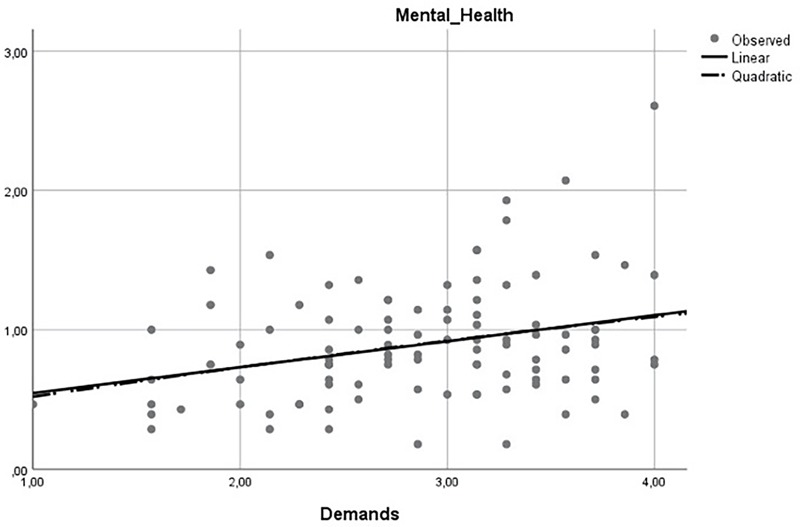
Linear and Curvilinear estimates for men.

The results of the linear and curvilinear estimates are represented in **Figures 2, 3**. Linear regression assumes that a straight line properly represents the relations between demands and the mental health. As can be observed in both figures, the linear model (represented by a continuous line) overlaps to the curvilinear model (represented by a dashed line). As such, there is low evidence for a curvilinear relationship between demands and mental health. At **Table 4**, the model summary and parameter estimates for the linear and curvilinear (quadratic) models are provided. Once again, we observe better indicators for the linear model [*women’s’ sample*: *F*(1,152) = 25.42, *p* < 0.01; *men’s sample*: *F*(1,98) = 9.54, *p* < 0.01] than for the curvilinear model [*women’s’ sample*: *F*(2,151) = 12.88, *p* < 0.01; *men’s sample*: *F*(2,97) = 4.73, *p* < 0.01] in both samples. Furthermore, through the hierarchical multiple regression analysis performed, we observed a significant link between demands and mental health at step one [*women’s’ sample*: *B* = 0.19, *p* < 0.01; *men’s sample*: *B* = 0.20, *p* < 0.01]. However, when demands squared (the quadratic term) was introduced at step two, once the linear relationship was controlled for in the same model, non-significant relationships were found between demands squared and mental health [*women’s’ sample*: *B* = -0.01, n.s.; *men’s sample*: *B* = 0.03, n.s.).

**Table 4:** Results for the linear and curvilinear (quadratic) regression models for the relationship between job demands and mental health.

Equation	*R*^2^	*F*	*df1*	*df2*	*p*	*b*_0_	*b*_1_	*b*_2_
Linear	(0.14) 0.09	(25.42) 9.54	(1) 1	(152) 98	(0.00) 0.00	(0.18) 0.36	(0.20) 0.19	
Quadratic	(0.15) 0.09	(12.88) 4.73	(2) 2	(151) 97	(0.00) 0.01	(0.36) 0.29	(0.05) 0.24	(0.03) -0.01

*The values presented on the right side of the brackets refer to the females; the values presented within brackets refers to the males.*

## Discussion

In this study, we examined an integrated and mediation moderated model in order to verify (1) the mediating role of the WFC in the relationship between job characteristics and workers’ mental health and (2) the role that gender plays as moderator of this relationship. The findings of our study seem to contribute to the idea that the WFC is important in explaining the relationship between job characteristics and workers’ mental health. Nevertheless, we also found that this relationship is not moderated by gender.

Regarding our mediation hypothesis, we observed different results concerning job characteristics. As expected, and in accordance with previous findings, we verified that both demands and control are potential important job characteristics for the emergence of the WFC (Grzywacz and Marks, 2000; Michel et al., 2011). This result reinforces the argument that job demands are a source of strain which depletes workers’ resources and evokes conflict in the family domain. On the contrary, job autonomy could prevent the WFC, since there is a possible negative contribution to the presence of the WFC. In fact, job demands and job autonomy are considered the core characteristics of the Job Demand-Control (Karasek, 1979). Furthermore, the WFC is significantly related to workers’ mental health, as also demonstrated by a number of studies (e.g., Berkman et al., 2015; Carvalho and Chambel, 2016; Neto et al., 2016). This finding seems to emphasize not only the crucial role that job characteristics play in the WFC but also the crucial role that the WFC has in explaining the relationship between job characteristics and workers’ mental health. However, the relationship between supervisory support and the WFC is not significant. This result may be related to the fact that we analyzed general social support and not work-family support specifically and, as stressed by the meta-analysis of Kossek et al. (2011), the work-family specific constructs of supervisory support [e.g., family supportive supervisor behavior (Hammer et al., 2009)] are more strongly related to the WFC. Another reason which may justify this finding could be the specific context of the organization where this study was conducted. In this organization, the work–family relationship is more dependent on organizational general rules and practices than on supervisory attitudes and actions.

Surprisingly, the present findings suggest that overall, men and women perceived the relationship between job characteristics and the WFC and the relationship between the WFC and their mental health equally. Considering the Portuguese context, in which dual-earner families are prevalent and traditional gender expectations subsist (Vieira et al., 2014), this finding reveals that the effects of work on Portuguese citizens are so critical, that gender does not excel. However, this unexpected result may be due to a number of different reasons. First, it is essential to underline the current Portuguese reality where, due to the economic crisis, Portuguese citizens are experiencing several austerity measures which have brought about increased job demands (e.g., longer working hours) (Ioakimidis et al., 2014) and job insecurity. In fact, Portuguese work values also point to security and stability as being crucial for workers (Chambel, 2013). Therefore, the impact of work on families’ lives is identical for men and women. Second, it may be possible for men and women to present identical results even though they differ in the way they manage their work and family life or in the way they use organizational solutions to deal with their work and family life. Third, despite the subsistence of gender expectations, it should also be noted that there are increasingly more egalitarian attitudes toward men and women across the world (e.g., Nohe et al., 2015) which, hopefully, will spread to Portugal in the near future. Finally, it is also necessary to draw attention to the fact that the mean age of the respondents was 52.83 years. Hence, we may deduce that the majority of respondents were not parents of young children, which is usually related to more family demands. Thus, for the respondents of this sample, particularly the women, family management may be easier. These hypotheses require further research in order to obtain a better understanding of the possible differences between men and women in their work–family relationship.

### Limitations, Future Studies, and Practical Implications

This study has some limitations that should be mentioned. First, the study used a self-report measure (questionnaire) that entails the problems of common method variance and consistency bias, however, as referred to by Spector (2006), common method variance concerns associated with heavy reliance on self-reported data measurements may be overstated. Second, analyses are based on a cross-sectional design. Hence, it is not possible to draw inferences regarding causal relations among the variables since the arrows that are depicted in **Figure 1** should be interpreted as associations. Thus, the causal ordering suggested by these results requires further confirmation in future longitudinal research. Third, this study was conducted in only one Portuguese organization which may compromise the generalizability of the results. Based on these limitations, future studies should continue to analyze our theoretical model. Moreover, as suggested by the meta-analysis of Kossek et al. (2011), the influence of work characteristics on the WFC depends on their relationship with this conflict. Hence, future studies should measure the family supportive supervisor behavior scale created by Hammer et al. (2009) and create job demands and autonomy scales that evoke the relationship with the family role. Furthermore, it is important to recognize that throughout the study we use the term ‘gender’ to mention the variable ‘biological sex’ that was used to measure male and female. Our option is solely related to the easy match between this construct and the literature on the WFC. However, the psychological implications of being male or female acknowledged in the gender construct are not integrated into this study. Thus, as stated by Powell and Greenhaus (2010), to fully understand the gender implications, future studies should include the measure of gender belief systems. Future studies should also include different demands using the Job Demands-Resources Model (Schaufeli and Bakker, 2004) and explore the possibility of curvilinear relations between different types of demands and workers’ outcomes.

In terms of theoretical and practical implications for the field of occupational health psychology, this study has built on prior research as it advances the WFC as a contributory mechanism to the explanation of the job characteristics and workers’ well-being relationship, as claimed by Schaufeli and Taris (2014). It should also be noted that the present study may provide some clues for the development of intervention agenda considering prevention of the WFC and, consequently, prevention of workers’ mental health deterioration. This study reinforces that job characteristics should be priorities in the future agenda. Furthermore, the results suggest that any intervention designed to reduce the WFC may have effective results for both men and women.

## Author Contributions

All the authors participated in the development of the article, giving suggestions and by making constant reviews. However, MN was more involved in the data collection, VC and MC were more involved in writing the article and SL in the statistical analysis.

## Conflict of Interest Statement

The authors declare that the research was conducted in the absence of any commercial or financial relationships that could be construed as a potential conflict of interest.
